# Identification of Cryptic *Anopheles* Mosquito Species by Molecular Protein Profiling

**DOI:** 10.1371/journal.pone.0057486

**Published:** 2013-02-28

**Authors:** Pie Müller, Valentin Pflüger, Matthias Wittwer, Dominik Ziegler, Fabrice Chandre, Frédéric Simard, Christian Lengeler

**Affiliations:** 1 Department of Medical Services and Diagnostic, Swiss Tropical and Public Health Institute, Basel, Switzerland; 2 University of Basel, Basel, Switzerland; 3 Mabritec AG, Riehen, Switzerland; 4 Spiez Laboratory, Swiss Federal Department of Defence, Spiez, Switzerland; 5 Maladies Infectieuses et Vecteurs: Ecologie, Génétique, Evolution et Contrôle, Institut de Recherche pour le Développement, Montpellier, France; 6 Department of Epidemiology and Public Health, Swiss Tropical and Public Health Institute, Basel, Switzerland; Centro de Pesquisas René Rachou, Brazil

## Abstract

Vector control is the mainstay of malaria control programmes. Successful vector control profoundly relies on accurate information on the target mosquito populations in order to choose the most appropriate intervention for a given mosquito species and to monitor its impact. An impediment to identify mosquito species is the existence of morphologically identical sibling species that play different roles in the transmission of pathogens and parasites. Currently PCR diagnostics are used to distinguish between sibling species. PCR based methods are, however, expensive, time-consuming and their development requires *a priori* DNA sequence information. Here, we evaluated an inexpensive molecular proteomics approach for *Anopheles* species: matrix assisted laser desorption/ionization time-of-flight mass spectrometry (MALDI-TOF MS). MALDI-TOF MS is a well developed protein profiling tool for the identification of microorganisms but so far has received little attention as a diagnostic tool in entomology. We measured MS spectra from specimens of 32 laboratory colonies and 2 field populations representing 12 *Anopheles* species including the *A. gambiae* species complex. An important step in the study was the advancement and implementation of a bioinformatics approach improving the resolution over previously applied cluster analysis. Borrowing tools for linear discriminant analysis from genomics, MALDI-TOF MS accurately identified taxonomically closely related mosquito species, including the separation between the M and S molecular forms of *A. gambiae* sensu stricto. The approach also classifies specimens from different laboratory colonies; hence proving also very promising for its use in colony authentication as part of quality assurance in laboratory studies. While being exceptionally accurate and robust, MALDI-TOF MS has several advantages over other typing methods, including simple sample preparation and short processing time. As the method does not require DNA sequence information, data can also be reviewed at any later stage for diagnostic or functional patterns without the need for re-designing and re-processing biological material.

## Introduction

Human malaria is exclusively transmitted by *Anopheles* spp. mosquitoes. Nearly all key malaria mosquito vectors – and many other mosquito species - are members of complexes or groups comprising morphologically indistinguishable sibling species [1]. Exact identification is, therefore, fundamental for understanding vector biology; and hence malaria risk factors and epidemiology. Equally, the success of vector control interventions profoundly relies on accurate information on mosquito populations to establish baseline data for the optimal choice of available tools and to monitor their effectiveness.

A widely discussed case is the *Anopheles gambiae* Giles 1902 species complex comprising at least seven morphologically identical sibling species across Africa [2]. Two of the members of this complex, *A. gambiae* sensu stricto (s.s.) and *A. arabiensis*, are major malaria vectors in sub-Saharan Africa and are found in sympatry over a large geographical range [3]. For malaria epidemiology and vector control an important aspect is the fact that these species differ in their biology. For example, *A. arabiensis* is more likely to rest outdoors for blood digestion making it a lesser target for indoor-residual spraying (IRS) with insecticides [4], [5]. Correct identification has even further-reaching practical consequences where a non-vector species is mistaken for a vector species and vice versa. In some areas, for instance, *A. arabiensis* is sympatric with *A. quadriannulatus* which - due to its strong preference to cattle [6], [7] - is generally considered an unimportant vector species. Moreover, sympatric mosquito species may show different levels of susceptibility to available insecticides for mosquito control (*e.g.* [8], [9]), thus underlining the importance of correct taxonomic classification. Even within *A. gambiae* s.s. further subdivisions are made. These subdivisions were initially defined by karyotypes and called “chromosomal forms” (*i.e.* Mopti, Bamako, Savanna, Bissau and Forest) [10]. The chromosomal forms can be further grouped into two genetically differentiated “molecular forms”, M and S, which were originally defined by variations in the intergenic spacer (IGS) and internal transcribed spacer (ITS) ribosomal DNA (rDNA) regions [11] and later found to be separated by other genetic associations [12], [13]. The M and S forms have been found to display different ecological tolerances and behaviours adding evidence of reproductive isolation between them [14]–[18].

The current “gold standard” to distinguish closely related and morphologically indistinguishable specimens is PCR diagnostics. Common to *Anopheles* mosquitoes are sequence variations within the second internal transcribed spacer (ITS2) of the rDNA providing valuable markers for diagnostic assays [1]. PCR protocols such as those developed to distinguish members of the *A. gambiae* complex (*e.g.* [19]–[23]) are important tools in basic and applied research. For routine screening they are, however, costly, time-consuming and labour intensive. Species specific PCR is also limited in its flexibility because primers target specific sequences and other potentially important markers may easily be overlooked.

To overcome the drawbacks of classic PCR-based methods, alternative methods have been explored more recently, notably loop-mediated isothermal amplification (LAMP) technique [24] and near-infrared spectroscopy (NIRS) [25], [26]. The LAMP technique is a DNA amplification process at a constant temperature using strand displacement reaction, allowing for amplification and detection of a gene in a single step. In contrast, NIRS collects a density distribution of the near-infra-red energy absorbed by a sample, which is then explored for characteristics that distinguish biological samples. Both methods may discriminate between the two closely related *A. gambiae* sibling species, *A. gambiae* s.s. and *A. arabiensis* and are potentially valuable methods in areas where the distinction between the two is sufficient [24]–[26].

A less explored avenue is the use of proteomic data as a taxonomic tool for insects. In contrast, characterisation and identification of microorganisms (*i.e.* bacteria and fungi) using whole cell matrix assisted laser desorption/ionization time-of-flight mass spectrometry (MALDI-TOF MS) is well established [27]. MALDI-TOF MS produces a mass spectrum that can be compared with reference spectra for rapid species identification and may be even more discriminating than rDNA sequence analysis [28]. Whole cell MALDI-TOF MS requires minimal sample preparation, has very low cost for consumables and produces results within minutes making it ideal for high throughput screening. Through improved hardware and advances in data storage for reference spectra and software solutions, MS has become a routine approach in the identification of prokaryotes [29], [30].

The use of MALDI-TOF MS for the discrimination of arthropod species has been evaluated for the first time almost a decade ago in fruit flies [31] and in aphids [32]. Since then the technique has received rather little attention among entomologists with a few recent exceptions. These include the study of Feltens *et al.* [33] in *Drosophila melanogaster*, by far the most in depth exploration of the method, Kaufmann *et al.* [34], [35] in biting midges and Karger *et al.* [36] in ticks.

The prospect of MALDI-TOF MS to discriminate arthropod species motivated us to set out and further explore this technique – together with a computational approach developed for “omics” data - for its use in malaria vector biology. Here, we measured MALDI-TOF MS spectra from 32 *Anopheles* laboratory colonies and 2 field populations representing 12 *Anopheles* species. The *Anopheles* colonies included key members of the *A. gambiae* species complex (*i.e. A. gambiae* s.s. of the M and S molecular form, *A. arabiensis*, the zoophagic *A. quadriannulatus* and one saltwater species, *A. merus*). This first account of successful implementation of MALDI-TOF MS to identify mosquito vectors of human disease will hopefully prove useful in the field and pave the way for many more related applications in vector biology and entomology.

## Materials and Methods

### Mosquitoes

All mosquito specimens used in the present study were female imagines (adults) with an age of at least two days post eclosure from the pupa. The specimens were either obtained from laboratory colonies or were collected in the field. Specimens from laboratory colonies (Table 1) were either sampled from our own colonies at the Swiss Tropical and Public Health Institute (Swiss TPH) and the Institut de Recherche pour le Développement (IRD; Montpellier and Burkina Faso) or obtained from the Malaria Research and Reference Reagent Center (MR4), VA USA. Field collections were carried out in West Africa in Ladji, Benin (6°21′10′’N, 2°24′30′’E) and Soumousso, Burkina Faso (11°01′46′’N, 4°02′45′’W, see [37]). In Benin, mosquitoes were sampled as larvae in the field and raised to imagines in the laboratory, whereas in Burkina Faso specimens were collected as resting females using aspirators inside human dwellings during the rainy season, in June 2010. In both cases, individual mosquitoes were morphologically identified as members of the *A. gambiae* species complex [38] and shipped to Swiss TPH in 70% ethanol. As found in biting midges [34] our preliminary tests showed that mosquitoes gave sufficient mass spectrometry (MS) signals even if kept for several months in ethanol (data not shown). Only the heads and thoraces were subjected to MS measurements as abdomens potentially introduce strong bias due to interference by remaining blood meals or changes in physiological status which might interfere with the overall signal – although we cannot fully exclude also some interference with *e.g. Plasmodium* parasites in the salivary glands. The abdomens were kept for molecular typing as described below.

**Table 1:** Laboratory colonies included in the MALDI-TOF MS analysis.

Species	Molecular form	Colony	Origin	Source^1^
Members of the *A. gambiae* species complex				
*A. quadriannulatus* s.s.		SKUQUA	South Africa	MR4 (MRA-761)
		SANGWE	South Africa	MR4
*A. merus*		OPHANSI	South Africa	MR4 (MRA-803)
		MAF	South Africa	MR4
*A. gambiae* s.s.	M	MALI-NIH	Mali	MR4 (MRA-860)
	M	MOPTI	Mali	MR4 (MRA-763)
	M	VK5	Burkina Faso	IRD
	M/S mix	VKPER	Benin	IRD
	M/S mix	RSP	Kenya	MR4 (MRA-334)
	S	G3	Gambia	MR4 (MRA-112)
	S	KISUMU1	Kenya	MR4 (MRA-762)
	S	PIMPERENA	Mali	MR4 (MRA-861)
	S	RSP-ST	Kenya	MR4 (MRA-698)
	S	SOUMOUSSO	Burkina Faso	IRD
	S	ZANU	Zanzibar	MR4 (MRA-594)
	S	IN22C+	Isolated from G3	MR4 (MRA-115)
	S (M/S hybrids in males)	ASEMBO1	Kenya	MR4 (MRA-186)
*A. arabiensis*		BOBO	Burkina Faso	IRD
		DONGOLA	Sudan	MR4 (MRA-856)
		HARARE	Mozambique	MR4
		KGB	Zimbabwe	MR4 (MRA-339)
		SENN	Sudan	MR4 (MRA-764)
Other *Anopheles* species				
*A. stephensi*		STE2	India	MR4 (MRA-128)
		STI	India	Swiss TPH
*A. quadrimaculatus*		ORLANDO	USA	MR4 (MRA-139)
*A. minimus*		MINIMUS1	Thailand	MR4 (MRA-729)
*A. freeborni*		F1	USA	MR4 (MRA-130)
*A. farauti*		FAR1	Papua New Guinea	MR4 (MRA-489)
*A. dirus*		WRAIR2	Thailand	MR4 (MRA-700)
*A. atroparvus*		EBRO	Spain	MR4 (MRA-493)
*A. albimanus*		STECLA	El Salvador	MR4 (MRA-126)
Outgroup				
*Aedes aegypti*		ROCK	North America	Swiss TPH

1 MR4: Malaria Research and Reference Reagent Center, VA, USA. Numbers in brackets are MR4 reference numbers; IRD: Institut de Recherche pour le Développement, Montpellier, France; Swiss TPH: Swiss Tropical and Public Health Institute, Basel, Switzerland. Numbers in brackets indicate the MR4 catalogue number.

### Molecular Typing

While the MR4 material was regarded as *bona fide*, IRD and Swiss TPH *A. gambiae* laboratory stock and field-caught specimens were genotyped according to the protocol of Wilkins *et al.* [20] with additional primers for simultaneous species and rDNA typing [39] using 1 µl of DNA extracted from the isolated abdomens. DNA was extracted from manually ground (plastic pestles) abdomens using the DNeasy® Blood & Tissue kit (Qiagen, Switzerland) according to the manufacturer’s protocol and eluted and stored in 200 µl Buffer AE. PCR products were loaded and run on a 2% agarose gel and visualised by ethidium bromide staining.

### MS Measurements and Data Pre-processing

For the MALDI-TOF MS, dissected head and thoraces were manually ground in a 1.5 ml Eppendorf tube containing 20 µl formic acid (10%). Five µl of the homogenate were then transferred into a new tube containing 7.5 µl saturated sinapic acid (SA) solution. SA solution consisted of 60% acetonitrile and 0.3% trifluoroacetic acid (Sigma-Aldrich, Switzerland). From the matrix suspension 4×1 µl were spotted on a custom made, 48 position steel target plate (Industrietechnik MAB AG, Basel, Switzerland) and air-dried.

**Figure 1:**
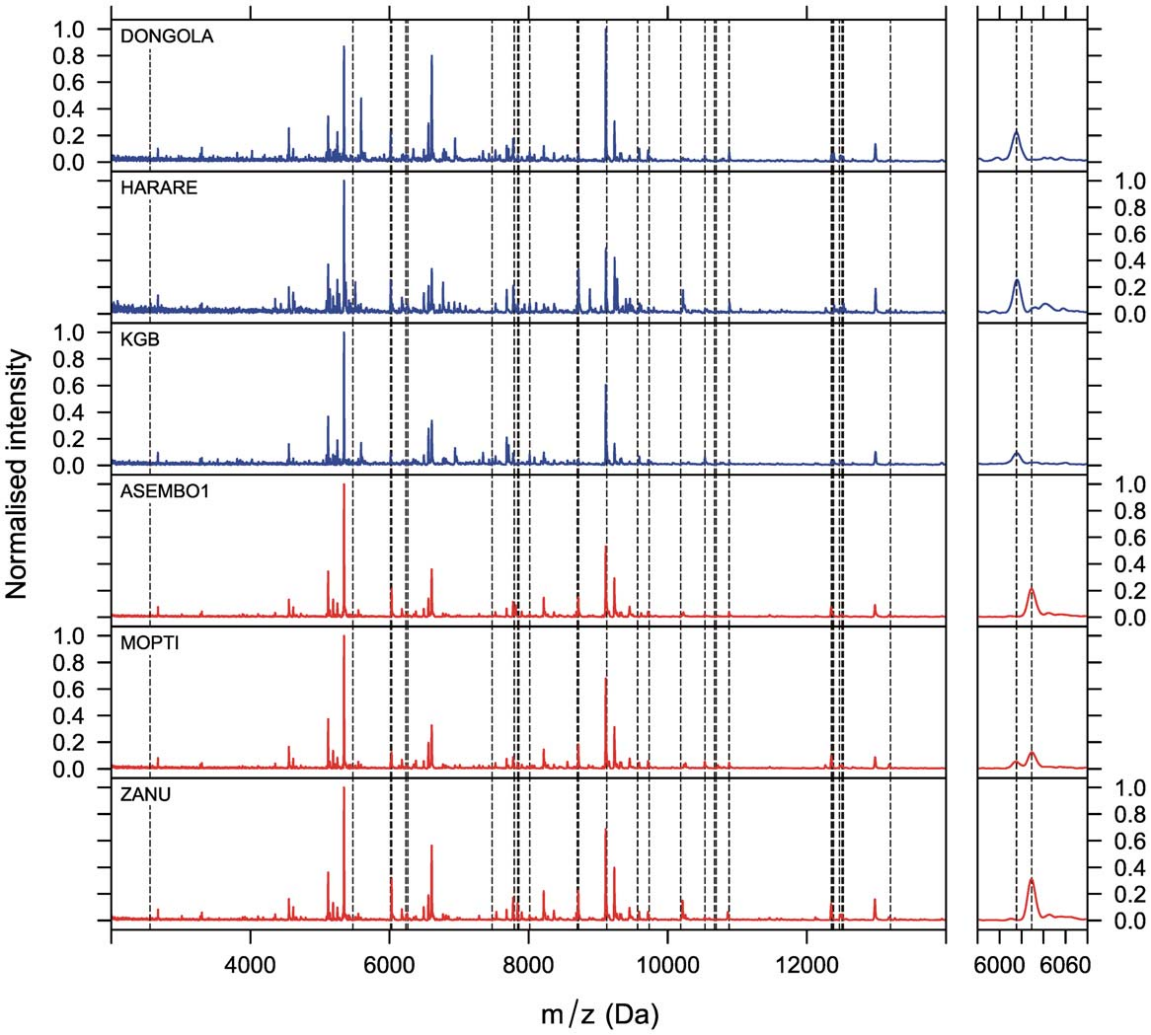
Examples of MALDI-TOF MS spectra for *Anopheles gambiae* sensu stricto and *A. arabiensis*. Examples of representative MALDI-TOF MS spectra measured from 3 *A. arabiensis* (blue) and 3 *A. gambiae* s.s. (red) colonies. The spectra were taken from crude suspensions of heads and thoraces in SA solution. The vertical, dashed lines indicate peaks that are characteristic (but not exclusive) for one or the other species. The left panels show the whole spectra between 2 and 14 kDa, while the right panels zoom into two peaks. The two peaks are separated by only a few Daltons. While the left peak is more common in *A. arabiensis*, the right peak is more common in *A. gambiae* s.s. In this representation the peak intensities were normalised against the highest intensity measured in each spectrum.

Raw spectra were acquired with an Axima™ Confidence MALDI-TOF mass spectrometer (Shimadzu-Biotech Corp., Kyoto, Japan) in the linear, positive mode over a *m/z* range of 2–30 kDa for a total of 1,000 laser shots per spotted sample. The machine’s parameters were chosen by setting the ion source at 20 kV and the extraction delay time at 200 ns. The spectra obtained by the spectrometer were then loaded into Launchpad™ 2.8 software (Shimadzu-Biotech Corp., Kyoto, Japan) to create a peak list for each sample spotted on the plate (*i.e.* four lists for each mosquito specimen). The peak lists with size (*m/z* value) and intensity values (arbitrary units) were then saved as ASCII text files for further data analysis. The peak lists and additional information are provided in Dataset S1 and Table S1. The software parameters were set to the following values: parent peak cleanup = “advanced scenario”; peak width = “80 chans”; smoothing filter width = “50 chans”; baseline filter width = “500 chans”; peak detection method = “threshold apex”. For the “threshold apex” peak detection, the threshold type was set as dynamic and the threshold offset to 0.02 mV with a response factor of 1.2. For external calibration purposes the software also used the spectra of the *Escherichia coli* DH5 alpha strain which was spotted alongside the mosquito samples on each 48-well plate. In addition, internal reference peaks that appear to be highly conserved in mosquitoes (*i.e. m/z* 2670.5, 4554.4, 5115.2, 5217.4, 5328.7, 5345.6, 5371.2, 5551.8, 5591.3, 6560.1, 7683.7, 8560.0, 9109.6, 9234.2, 9453.3, 10255.6 and 16724.8, see also results Figure 1) were used for internal calibration within a range of ±700 ppm.

### Unsupervised Cluster Analysis

For the unsupervised cluster analysis, the peak lists, previously saved as ASCII text files, were loaded into SARAMIS™ 3.4.1.11 (AnagnosTec, Germany) to create a binary presence/absence table for each peak in the whole data set with columns for peaks and rows for spectra. Peaks were binned within ±800 ppm. A custom-written macro in Excel 2002 (Microsoft) then merged the peak lists into a single “average” peak list for each mosquito specimen. Here, a peak was deemed present if it was detected in at least three out of four lists. The consolidated table was then loaded into the freely available PAST 2.14 software and a dendrogram generated using the “Dice” multivariate clustering algorithm for paired groups [40]. The computed output was exported in nexus file format and displayed with the freely available FigTree 1.3.1 programme (available from http://tree.bio.ed.ac.uk) and labels adjusted for visibility with CorelDRAW 12 (Corel Corporation, 2003).

### Supervised Linear Discriminant Analysis

In addition to the unsupervised clustering approach described above we have also taken a supervised approach using linear discriminant analysis (LDA), an extension to the approach applied to bacteria by Wittwer et al. [41], to find combinations of features (*i.e.* peaks) that separate the “taxonomic” groups at three levels:

Model 1: Classify between members of the *A. gambiae* species complex;

Model 2: Classify *A. gambiae* s.s. into M and S molecular forms; and

Model 3: Classify laboratory colonies of the same *Anopheles* species.

For constructing the LDA model we have chosen the shrinkage discriminant analysis (SDA) procedure described in Ahdesmäki and Strimmer [42] because it addresses well the “small *n*, large *p*” (*i.e.* few data points, many features) issue and correlation between peaks, a typical feature of MS spectra. Here, training of the classifier is based on James–Stein shrinkage estimates of correlations and variances, where regularisation parameters are chosen analytically without re-sampling and therefore computationally non-intensive. The approach applies a pooled centroids formulation of the multiclass LDA predictor function, in which the relative weights of Mahalanobis-transformed predictors are given by correlation-adjusted *t*-scores (CAT scores). The CAT scores allow for simple ranking and selection of peaks.

For the supervised LDA, ASCII files containing the peak list for each measurement (*i.e.* four files per specimen) were imported into the open source statistical software package R version 2.14.1 [43]. The peaks from the original peak lists were dynamically binned with the R package “caMaClass” [44], with a variable bin size between 800–1600 ppm. Here, dynamical binning means that all spectra (*i.e.* peak lists) were aligned to account for small offsets between peak maxima that would still represent the same peak. Peak intensities were log_10_-transformed and then – in line with the unsupervised clustering approach - averaged across the four spectra from the same individual mosquitoes if a signal was present in at least three out of the four spectra. The functions in the R package “sda” [45] were then applied for the SDA as described in Ahdesmäki and Strimmer [42].

For all three models, only laboratory colonies relevant to the specific taxonomic problem were included (Table 2). To build and to test the predictive performance of the SDA model the data was randomly split into two sets so that spectra from five specimens per colony served as the training and validation set for furnishing the SDA classification model and the spectra from the other five mosquitoes per colony formed the true test set for estimating the generalised classification error of the model.

**Table 2:** Laboratory colonies included to build the shrinkage discriminant analysis (SDA) models.

Colony	Species	Molecular form	Model 1^1^	Model 2^2^	Model 3^3^
SANGWE	*A. quadriannulatus* s.s.	-	x		
SKUQUA		-	x		
MAF	*A. merus*	-	x		
OPHANSI		-	x		
ASEMBO1	*A. gambiae* s.s.	S	x	x	
G3		S	x	x	
IN22C+		S	x	x	
KISUMU1		S	x	x	
MALI-NIH		M	x	x	
MOPTI		M	x	x	
PIMPERENA		S	x	x	
RSP		M/S mix	x		
RSP-ST		S	x	x	
SOUMOUSSO		S	x	x	
VK5		M	x	x	
VKPER		M/S mix	x		
ZANU		S	x	x	
BOBO	*A. arabiensis*	-	x		x
DONGOLA		-	x		x
HARARE		-	x		x
KGB		-	x		x
SENN		-	x		x

1 Model 1: SDA model to discriminate between members of the *A. gambiae* species complex.

2 Model 2: SDA model to discriminate between M and S molecular forms within *A. gambiae* s.s.

3 Model 3: SDA model to classify specimens to their colony of a single species, *i.e. A. arabiensis*.

Our approach was to minimise the number of the selected peaks in an attempt to avoid overfitting. The minimum number of peaks was iteratively determined by adding additional peaks with decreasing CAT scores in a stepwise manner to the model, while estimating the error rate from the cross-validation of each set of predictors. The error rate was calculated as the number of failed classifications over the total number of successful plus failed classifications committed over 1,000 iterations whereby in every iteration step, a single record from each group (*e.g.* one from each *A. gambiae* sibling species) of the complete training set was removed by chance and the rest used to fit the classification model.

The final classification model was eventually challenged by its application to the specimens that were not used to build and cross-validate the model in order to measure the generalised classification error rate.

## Results and Discussion

In total, we recorded spectra from 320 laboratory specimens, including 32 *Anopheles* colonies and one *Aedes aegypti* colony (Table 1), and spectra from 125 field-caught specimens that included a mixture of sibling species and molecular forms from two field populations, “Soumousso” in Burkina Faso (20 *A. arabiensis*, 35 *A. gambiae* s.s. M form and 51 *A. gambiae* s.s. S form) and “Ladji” in Benin (19 *A. gambiae* s.s. M form). MALDI-TOF MS spectra measured from heads and thoraces suspended in SA matrix solution produced peaks in the raw spectra with *m/z* values ranging between 2 and 29.8 kDa with the majority (95% of all peaks) lying within 2 and 15.7 kDa. On average, 121 peaks were detected in a single raw spectrum, ranging between 48 and 187 peaks. This is in the range of MS spectra previously obtained from biting midges using a similar approach [34]. Given that the *A. gambiae* proteome encompasses approximately 13,000 proteins [46] the number of peaks acquired with the current method suggests that only a very limited fraction of the whole proteome is represented. Despite the weak representation of the proteome some peaks appeared to be more characteristic – but not exclusive markers - for certain species even by visual inspection of the unprocessed MS spectra (Figure 1). Intriguingly, in some instances the peaks were separated by only a few Daltons, perhaps reflecting single amino acid substitutions or minor post-transcriptional modifications. Indeed, such small differences were previously found in orthologous neuropeptides from different *Drosophila* species by MALDI-TOF MS [47].

### Unsupervised Cluster Analysis

Previous studies that evaluated the feasibility of using whole cell MALDI-TOF MS to distinguish between arthropod species either identified distinct patterns, with similarity within and differences between species, by visual examination [31], [32] or hierarchical cluster analysis [33], [34], [36].

**Figure 2:**
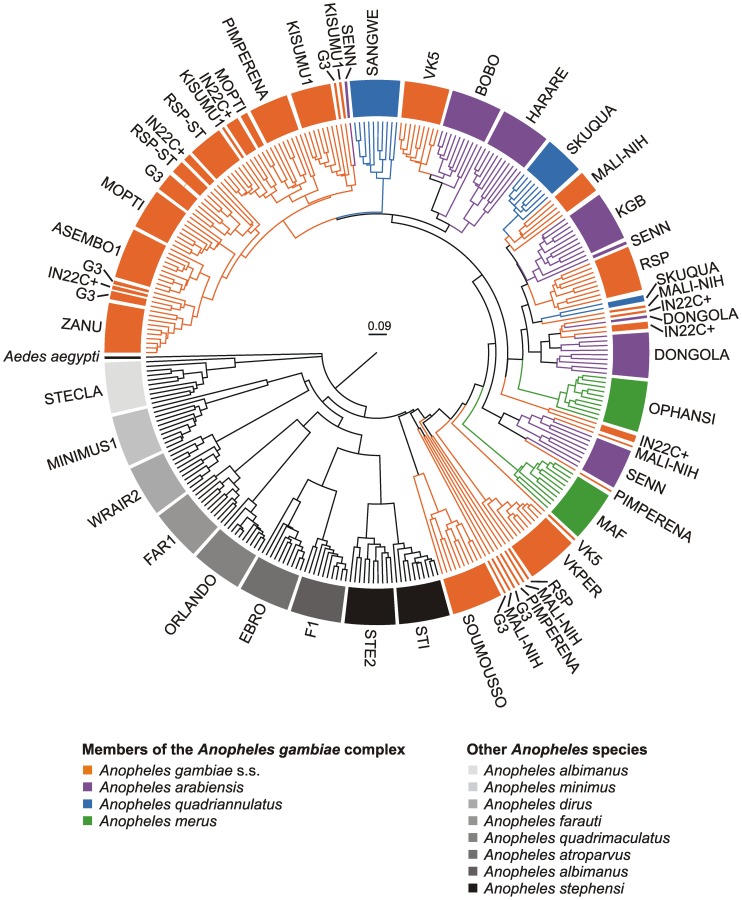
Dendrogram of hierarchical, unsupervised clustering of binary peaks (presence/absence). While the *Anopheles* species (complexes) are well separated by the cluster algorithm, the sibling species of the *A. gambiae* complex (coloured lines) do not segregate into well defined clusters. Specimens, both from the same species and colony, are split into different groups. The external branches represent each measured specimen. For each colony spectra from 10 specimens were recorded and included in the cluster analysis. The labels give the names of the colonies (Table 1). The length of the branches corresponds to the size of the Dice similarity coefficient.

Initially we also set out to use a cluster analysis approach. At first inspection, colonies from the same species (complex) that were reared in different laboratories over many years clustered well together into the same super cluster (Figure 2). A good example is *A. stephensi*. The individual specimens form the two colonies included in this study segregate into two clusters and yet aggregate into one single cluster for that species at the next higher level. This is in contrast to the *A. gambiae* species complex where hierarchical clustering failed to segregate the (four analysed) sibling species within the complex (Figure 2). Conceivably, the intermixture within the species complex mirrors the close relationship among the *A. gambiae* sibling species. In line with the lack of distinct hierarchical clusters there were no unique peaks that would serve as single biomarkers to separate the sibling species.

### Sibling Species Classification within the *Anopheles gambiae* Complex (Model 1)

**Figure 3:**
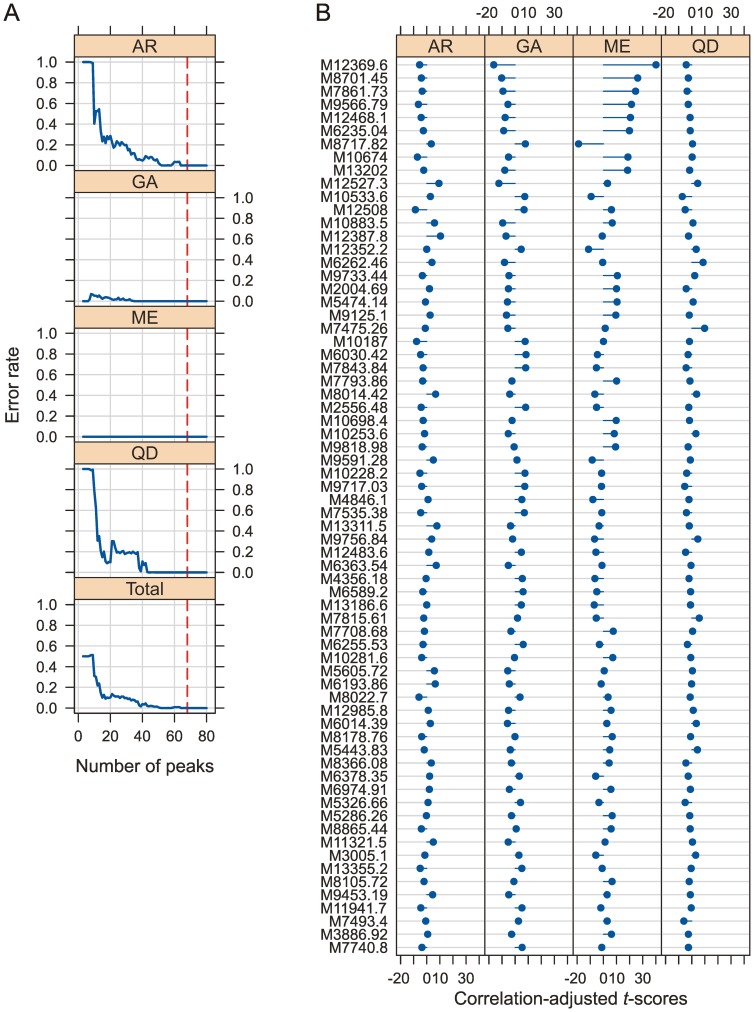
Model selection and cross-validation to discriminate between species of the *Anopheles gambiae* complex (Model 1). (**A**) The graph shows the error rate from the cross-validation plotted as a function of the number of the ranked peaks included in the SDA model that discriminates between members of the *A. gambiae* species complex. The peaks were ranked (left to right) according to the correlation-adjusted *t*-scores (CAT scores). The vertical, red line shows the 68 peaks chosen for the SDA model. (**B**) List with the 68 ranked peaks (top equals highest rank) their corresponding CAT scores. The length and direction of the horizontal blue bars represents the CAT scores of the centroid versus the pooled mean and show the influence of a particular peak in differentiating between the groups (Table S2). For example the top peak, M12369.6 has a strong influence in separating *A. merus* from all the other species, emphasised by the length of the bar and the opposite direction from the bars of the other species. In contrast, the tenth peak, M12527.3 has a stronger influence in separating *A. gambiae* s.s. from *A. arabiensis*. AR: *A. arabiensis*; GA: *A. gambiae* s.s.; ME: *A. merus*; QD: *A. quadriannulatus*.

In an attempt to overcome the poor performance of the unsupervised cluster analysis in discriminating between the *A. gambiae* sibling species, a SDA classification model was evaluated as an alternative. The model (Model 1) was trained using 110 specimens, 5 individual mosquitoes from each of 22 laboratory colonies including 5 *A. arabiensis*, 13 *A. gambiae* s.s., 2 *A. merus* and 2 *A. quadriannulatus* colonies (Table 2). When ranked by the CAT scores, including the top 68 peaks gave a model with zero remaining total error rate in the cross-validation (Figure 3 and Table S2). For estimating the generalised classification error of the final model the other 110 specimens, not used for model building and cross-validation, from the laboratory colonies plus an additional set of 125 field-caught female mosquitoes were classified using Model 1.

Model 1 correctly classified 105 out of 110 laboratory specimens (95% accuracy), while the model’s performance for the field-caught mosquitoes was lower with 105 out of 125 specimens correctly identified (84% accuracy). Taking a closer look at the field specimens it turns out that the 19 specimens reared from field caught larvae were all accurately classified, while only 86 out of 106 specimens (*i.e.* 81%) caught by aspiration were correctly identified. Altogether this means that pooling the specimens that were processed in the same way (*i.e.* raised from larvae) show an astonishing accuracy of 96% (124 out of 129). PCR and MALDI-TOF MS scores for each specimen included in the analysis are provided in Table S1.

**Figure 4:**
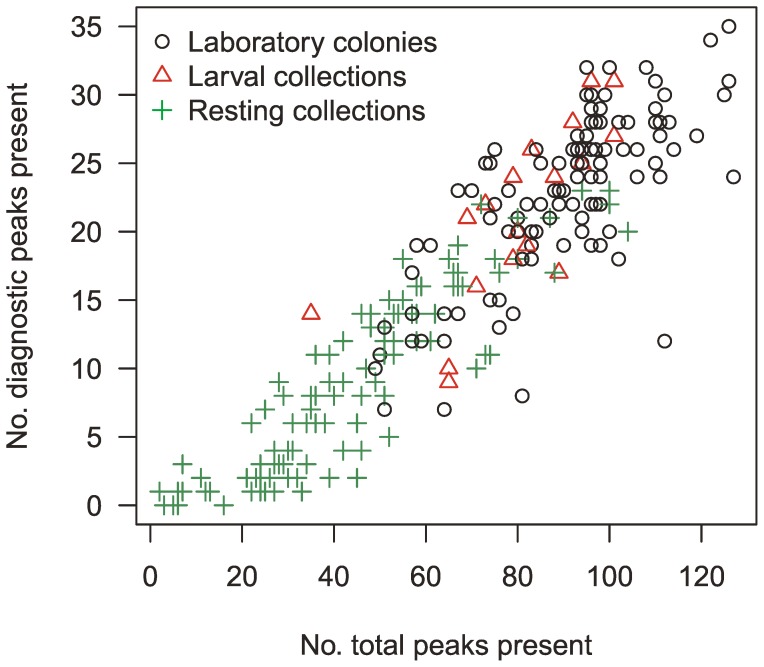
Total number of peaks versus number of diagnostic peaks present in average spectra from the *Anopheles gambiae* species complex. The number of diagnostic peaks present is associated with the number of total peaks present in an average peak list. The diagnostic peaks refer to the 68 selected peaks to distinguish within the *A. gambiae* species complex (Model 1). The plot suggests that the field specimens collected by aspiration (green crosses) were generally of lower quality (*i.e.* showing fewer peaks) than the specimens that were raised from the larvae; regardless whether from laboratory (black circles) or field caught larvae (red triangles).

There might be many – biological and technical – reasons why the specimens from the resting collections performed less well in the MALDI-TOF MS analysis. The specimens – unlike the adults raised from the larval collections – were either blood fed or gravid females or perhaps even carrying pathogens. Therefore, it is expected that a subset of genes would be differentially expressed due to the physiological state of the mosquito (*e.g.* [48]–[50]). Such differential expression would potentially also affect the number and types of masses detected in the MALDI-TOF spectra. If this was the prime cause of poor performance of Model 1 on the specimens from the resting collections we would predict different masses to come up in the MS spectra. The observation in our data set was, however, a different one. The number of peaks obtained from these specimens was lower than from those raised from larvae. More importantly, the number of diagnostic peaks present in an average spectrum showed the same relationship for all types of samples (Figure 4). It is, therefore, concluded that a major impact on the poorer performance of Model 1 on these specimens is a quality rather than a biological phenomenon. Indeed, some of the spectra even showed zero peaks (Figure 4). Including the spectra from field specimens in the model did also not improve its performance (data not shown). Perhaps the blood in the abdomens somehow negatively influenced the preservation of the specimens from those resting collections. Although somewhat unfortunate for the current study this is an aspect that can be addressed by optimising and standardising sampling, storage and processing procedures in future studies. A preliminary recommendation would be to separate the abdomens from the head and thoraces prior storage.

### Biological Meaning of the Detected Peaks

An interesting question would be what peptides or proteins the selected peaks actually represent and whether the observed patterns have any biological meaning, but without thorough additional investigation and access to genetic information on the different mosquito taxa the masses themselves only lend to speculation.

Feltens *et al.* [33] investigated some proteins that came up in MALDI-TOF MS profiling in *D. melanogaster* using nano-high-performance liquid chromatography electro spray ionisation tandem MS. Most of them were identified as originating from muscle tissues and mitochondria. As mentioned above, a caveat underlying the MALDI-TOF MS is that the full complement of proteins and peptides cannot be detected. Similarly, it might also miss out on detecting differences between epicuticular lipid profiles that have been described between *A. gambiae* M and S molecular forms as well as *A. arabiensis* as they are below the detection range [51].

### Classification of *Anopheles gambiae* Sensu Stricto M and S Molecular forms (Model 2)

**Figure 5:**
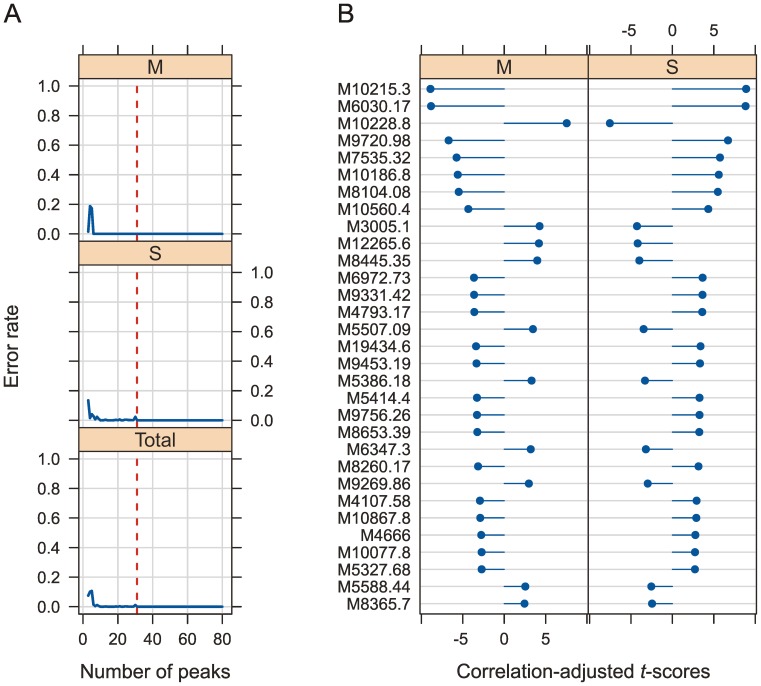
Model selection and cross-validation to distinguish molecular M and S forms in *Anopheles gambiae* sensu stricto (Model 2). (**A**) Error rate of the cross-validation plotted as a function of the number of the ranked peaks included in the SDA Model 2 that classifies M and S molecular forms among *A. gambiae* s.s. The peaks were ranked according to the correlation-adjusted *t*-scores (CAT scores). The vertical, red line shows 31 chosen peaks for the SDA model. (**B**) The 31 peaks listed on the left were selected on the basis of the smallest number of peaks still providing the lowest error rate shown in (A) and were ranked according to their CAT scores (Table S3). The length and direction of the horizontal blue bars represents the CAT scores of the centroid versus the pooled mean and shows the influence of a particular peak in differentiating between the two molecular forms. M: *A. gambiae* s.s. molecular M form; S: *A. gambiae* s.s. molecular S form.

Remarkably, by using the SDA approach it was possible to come up with a SDA model (Model 2) that allows for discriminating between the M and S molecular forms of *A. gambiae* s.s. (Figure 5 and Table S3). Among the 11 laboratory colonies (8 S and 3 M form colonies; Table 2) 50 out of 55 (91%) individuals that were not used to build the model were still correctly identified as either M or S form with 31 peaks in the model (Table S1).

The model failed to discriminate between the M and S molecular forms in the field caught specimens (classification error rate equals 49%). While there were some quality issues as discussed above, additional discrepancies between the rDNA typing method and MALDI-TOF might have arisen due to non-interchangeability between methods as found between several rDNA methods [52], [21]. It also appears that the discriminating pattern obtained from the laboratory specimens does not well represent the field caught mosquitoes. This may likely be overcome by adding field specimens into the reference set for building the SDA model.

Although the CAT scores are no direct measurement of phylogenetic distances the computed values qualitatively match the expectation that distances between higher taxa would generally be greater than those of closely related taxa. When comparing the range of CAT scores in Figure 3b (Model 1; discriminating between *A. gambiae* sibling species) to those in Figure 5b (Model 2; discriminating molecular M and S molecular forms) the observed patterns actually meet that prediction.

### Classification of *Anopheles arabiensis* Laboratory Colonies (Model 3)

**Figure 6:**
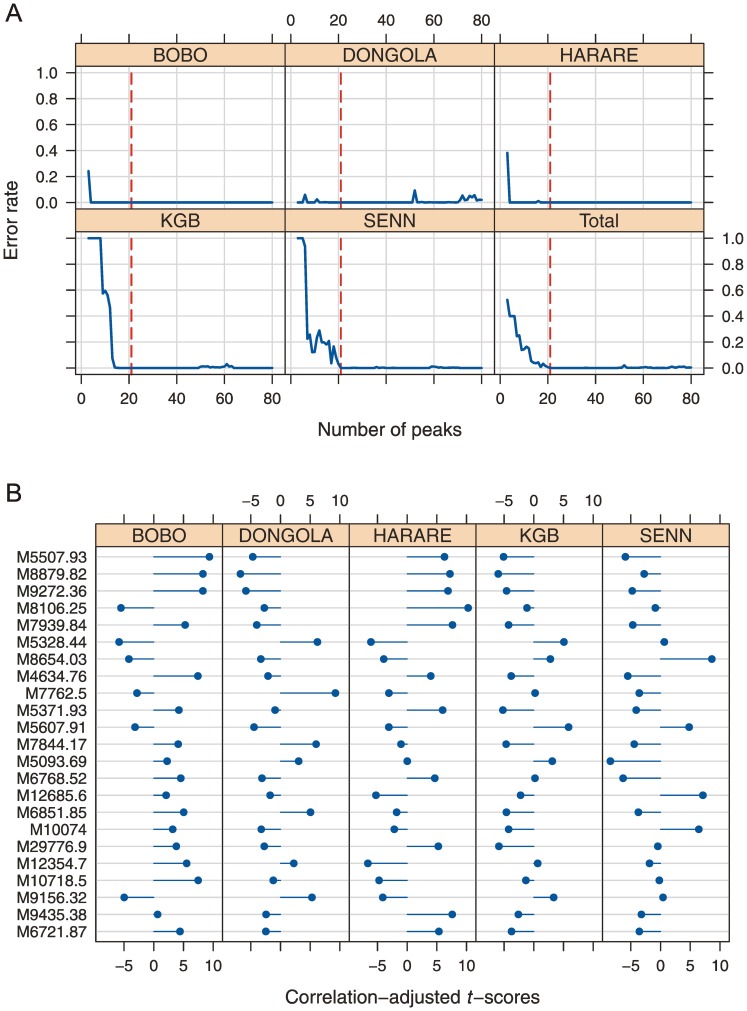
Model selection and cross-validation for colony authentication in *Anopheles arabiensis* (Model 3). (**A**) Error rate as a function of the number of peaks included in the SDA model for five *A. arabiensis* colonies and the total error rate over all colonies. The peaks were ranked according to the correlation-adjusted *t*-scores (CAT scores). The vertical, red line shows the 23 peaks chosen for the SDA model (Table S4). (**B**) Top 23 peaks included in SDA model after they were ranked according to CAT scores (*i.e.* peak with highest CAT score appears at the top of the list). The length and direction of the horizontal blue bars represents the CAT scores of the centroid versus the pooled mean and shows the influence of a particular peak in differentiating between the colonies.

The SDA Model 3 (Figure 6 and Table S4), classifying specimens of the same species into their colonies of origin, accurately scored 20 out of 25 specimens (80%) among the five *A. arabiensis* laboratory colonies (Table S1). Though 80% accuracy may seem low this is still quite remarkable given that the model is based on only 5 randomly picked individuals per colony. Including more features than the minimum 21 peaks yielded by our inclusion criteria would actually increase accuracy even more. For example, including an additional 7 peaks into the model provides an accuracy of 88% (*i.e.* 22 correctly identified out of 25 individuals). Including more specimens in the training set would also reduce the classification error (data not shown).

### How does the Approach Compare to Other Species Diagnostic Tools?

Assays widely used to distinguish between members of the *A. gambiae* species complex are PCR diagnostics based on sequence variations within the ITS2 of the rDNA [53], [19]–[21]. These assays require several steps in processing mosquito specimens; DNA extraction, PCR amplification and finally visualisation of the amplicon. Altogether these steps are labour and cost intensive. Even just the consumables for a single extraction followed by a PCR step can easily be 100 times more expensive than the consumables for a crude MALDI-TOF measurement - not even mentioning the increased labour costs and processing time.

An obstacle for the use of MALDI-TOF MS, particularly in disease endemic countries could be the large capital outlay required for acquiring and running a spectrometer and the need for specially trained personnel. Once a sound data basis and the analytical tools are implemented into automated systems this technique may, however, become an accessible tool for a wider community and a valuable alternative for large scale screening programmes.

For smaller projects an interesting alternative is LAMP, loop-mediated isothermal amplification technique as it uses little laboratory equipment and is much faster than current PCR methods [24]. The method, however, still requires knowledge of species specific differences to develop the assay and design of sequence specific primers in the case of multiple species is challenging.

MALDI-TOF MS has the great advantage that *a priori* knowledge of sequence variations is not needed and once spectra are acquired data can be revisited at any time *in silico* as already demonstrated here. For example, separating *A. arabiensis* from *A. gambiae* s.s. the same data could be used to first separate between the species and then to further classify *A. gambiae* s.s. into M and S molecular forms. Similarly this would allow for re-running the analysis should taxonomy change in one or the other way.

Another approach that has been proposed to discriminate between *A. gambiae* s.s. and *A. arabiensis* specimens is NIRS, near infrared spectroscopy [25]. Similar to our approach spectra are recorded and explored for discriminant patterns. The spectra themselves are, however, less conducive in drawing conclusion as to what causes them and what makes the differences between classes due to the complex nature of the spectra. An association between observed patterns and specific chemical components is extremely difficult if not impossible.

In summary, combining acquisition of MALDI-TOF MS spectra and statistical analytical tools to classify mosquito specimens appears very promising for *Anopheles* research and routine surveys for vector control programmes and, most importantly, entomology in general. A strength of the SDA algorithm is also that it takes into account peak intensities adding to the possibilities in discriminating patterns. The taxonomic classes are separated by patterns rather than single diagnostic peaks. It is expected that models including more reference specimens together with better storage and/or processing procedures will increase accuracy and add further value to this technology “repurposed” from microbiology. Furthermore, purification of the protein extracts might yield larger numbers of peaks similar to those found in *D. melanogaster* [33] that would also allow for investigating the nature of the peaks themselves.

### Conclusions

The present study shows that MALDI-TOF MS reliably discriminates between anopheline mosquito species - even at the sub-species level. Present data suggests that even colony-specific patterns are resolved and that the technique may be used beyond simple species typing including stock authentication or perhaps the detection of population structures in field-caught mosquitoes. While being accurate and robust MALDI-TOF MS has several additional advantages over other typing methods, including simple sample preparation, short processing time and low consumable costs – providing results rapidly and economically. The workflow can easily be standardised and automated allowing for cost-effective high throughput mass screening. As the method does not require DNA sequence information about the mosquito, data can be reviewed at any later stage for diagnostic or functional patterns. As only parts of the animal are needed the remaining parts can be subjected to additional analysis on DNA or protein extracts of the same individual. This method has the potential to become an invaluable tool for many applications in vector biology and control including routine species identification, colony authentication, population genetics or even the detection of trait-specific markers including insecticide resistance. These and other possibilities are currently being further explored in our laboratories.

## Supporting Information

Table S1 **MALDI-TOF MS classification results.** The table shows the specimens included in the three different models and whether the models classified the individuals correctly (TRUE) or wrongly (FALSE) against the reference (i.e. morphology, PCR score and information provided by MR4). PEAKLIST: file name of the MALDI-TOF MS peak list. LABEL: name of the mosquito colony or the field population. MR4.ID: MR4 catalogue number. ORIGIN: tells whether the specimen was collected in the field or originating from a laboratory colony. SPECIMEN: is the specimen number of a colony (1 through to 10) or field population. SPECIES: mosquito species. MOL.FORM: molecular form in the case of *A. gambiae* s.s. INCLUSION.MODEL.1: indicates whether the spectrum/specimen was included in building Model 1. CLASSIFICATION.MODEL.1: states whether the specimen was classified correctly (TRUE), wrongly (FALSE) or was not included in the classification (NA). INCLUSION.MODEL.2: same as above for Model 2. CLASSIFICATION.MODEL.2: same as above for Model 2. INCLUSION.MODEL.3: same as above for Model 3. CLASSIFICATION.MODEL.3: same as above for Model 3.
(CSV)

Table S2 **CAT scores for the SDA model discriminating the** ***Anopheles gambiae*** **species complex (Model 1).** The table lists the computed correlation-adjusted *t*-scores (CAT scores) of the mean versus the pooled mean for each predictor variable (*i.e.* peak) and centroid. Score: The sum of the squared CAT scores across groups which determines the overall ranking of the peaks. CAT.AR, CAT.GA, CAT.ME and CAT.QD are the CAT scores of the centroid versus the pooled mean for each group and peak. LFDR: The local false discovery rate computed for each peak. HC: The higher criticism score computed for each peak.
(CSV)

Table S3 **CAT scores for the SDA model discriminating M and S molecular forms in** ***Anopheles gambiae*** **s.s. (Model 2).** The table lists the computed correlation-adjusted *t*-scores (CAT scores) of the mean versus the pooled mean for each predictor variable (*i.e.* peak) and centroid. Score: The sum of the squared CAT scores across groups which determines the overall ranking of the peaks. CAT.M and CAT.S are the CAT scores of the centroid versus the pooled mean for each group and peak. LFDR: The local false discovery rate computed for each peak. HC: The higher criticism score computed for each peak.
(XLS)

Table S4 **CAT scores for the SDA model discriminating the** ***Anopheles arabiensis*** **colonies (Model 3).** The table lists the computed correlation-adjusted *t*-scores (CAT scores) of the mean versus the pooled mean for each predictor variable (*i.e.* peak) and centroid. Score: The sum of the squared CAT scores across groups which determines the overall ranking of the peaks. CAT.BOBO, CAT.DONGOLA, CAT.HARARE, CAT.KGB and CAT.SENN are the CAT scores of the centroid versus the pooled mean for each group and peak. LFDR: The local false discovery rate computed for each peak. HC: The higher criticism score computed for each peak.
(XLS)

Dataset S1 **MALDI-TOF MS peak lists.** The folder contains the 1,740 MALDI-TOF MS peak lists that were the basis of the present analysis. The file names correspond to the column “PEAKLIST” in Table S1 containing a detailed description of the specimens and its classification by the different SDA models.
(ZIP)
